# Transungual Delivery of Ciclopirox Is Increased 3–4-Fold by Mechanical Fenestration of Human Nail Plate in an In Vitro Model

**DOI:** 10.3390/pharmaceutics11010029

**Published:** 2019-01-14

**Authors:** Damian Cordoba Díaz, Marta Elena Losa Iglesias, Ricardo Becerro de Bengoa Vallejo, Manuel Cordoba Diaz

**Affiliations:** 1 Departamento de Farmacia y Tecnología Farmacéutica, Facultad de Farmacia, Universidad Complutense de Madrid, 28040 Madrid, Spain; damianco@farm.ucm.es (D.C.D.); mcordoba@farm.ucm.es (M.C.D.); 2 Facultad Ciencias de la Salud, Universidad Rey Juan Carlos, 28933 Madrid, Spain; 3 Facultad de Enfermería, Fisioterapia y Podología, Universidad Complutense de Madrid, 28040 Madrid, Spain; ribebeva@ucm.es

**Keywords:** transungual, onychomycosis, ciclopirox, fenestration, topical, antifungal

## Abstract

Onychomycosis is a fungal infection of nails that is widespread and difficult to treat because of the impermeable nature of human nails. Topically applied anti-fungal agents cannot penetrate this structure, and treatment regimens often resort to systemic antifungals with concomitant side effects. One recent clinical study suggested that mechanical fenestration of the nail using an intelligent nail drill might be a possible solution to this problem. In this work, an in vitro model of the transungual delivery of antifungal agents is presented, which utilizes real nail tissue and an inline flow system. This system was deployed to measure transungual delivery of ciclopirox and determined that nail fenestration improved drug delivery by 3–4-fold after 42 days. This study bolsters the argument that nail fenestration should be accepted as a pretreatment for onychomycosis and offers a way of evaluating new drugs or formulations designed to combat this condition.

## 1. Introduction

Onychomycosis is a fungal infection of toenails and fingernails [1]. It most commonly affects the nails of the hallux and is more prevalent in men than women, which is possibly because the male hallux nail is generally thicker than the female nail (1.68 mm versus 1.38 mm) [2]. Both systemic and topical antifungal agents are used as treatments for onychomycosis [1,2,3]. The efficacy of topically applied antifungal agents is hampered by the need for the agent to pass through the human nail [4].

The nail plate is composed of three layers: the dorsal–superficial, intermediate, and ventral layers [5]. The ventral nail plate and the subungual keratin in the nail bed are the layers that are most likely to be infected by fungi, such as *Trichophyton rubrum* and *T. mentagrophytes* [6]. The upper (dorsal) layer constitutes the main barrier to drug diffusion through the nail plate despite having a thickness of only a few cell layers [6,7]. When a drug is applied topically, it can accumulate in the dorsal nail surface layer and display very limited diffusion into the deep ventral layer and the bed [6,7]. As a result, the concentration of an applied drug can drop ~1000-fold across the width of the nail and fail to reach the minimum inhibitory concentration (MIC) needed to arrest fungal growth [5]. 

The dorsal layer, therefore, limits the number of antifungal drugs that can be used to treat this condition since any potential agent must possess good permeability (i.e., small molecular weight, hydrophilicity, and low keratin affinity) and must also be soluble in a suitable carrier formulation so that it can be carried across the dorsal surface into the ventral layers [4].

One possible solution to this problem was recently presented. This involves mechanical perforation or fenestration of the nail using an intelligent nail drill that is able to penetrate the nail plate without damaging the delicate nail bed beneath [8]. One study used this technique to treat three cases of onychomycosis using topically applied antifungal agents [8].

Despite the promise shown by mechanical fenestration as a clinical tool, there is currently no model system described that quantitatively measures transungual delivery of topically applied antifungal agents in fenestrated nails. This study aimed to develop such a model system and use it to measure the delivery of ciclopirox to the nail bed and ventral layers using an in vitro finite dose model.

The results described in this present study should help evaluate the effect of mechanical fenestration on the permeability of topical formulations and determine the best agents to treat onychomycosis prior to the start of clinical trials.

## 2. Materials and Methods

Approval for this study was provided by the Research Committee of the Hospital Clínico San Carlos de Madrid (18/448-E).

Twelve healthy human hallux toenails were collected from six adult human cadavers of Caucasian descent that were supplied by the Anatomy Laboratory at the Complutense University of Madrid—Spain) as previously described in a similar study [9]. All cadavers were male in order to minimize the variation in nail thickness, since this trait is known to show gender-specific variation [2]. All hallux nails were of similar shape and morphology. Any malformed hallux nails were rejected because distortions in structure or shape might have presented an uncontrolled source of variation. Diseased nails were also discarded for the same reasons. Twelve nails were chosen, as this is the minimum number of subjects required by the Committee for Medicinal Products for Human Use (CHMP) guidelines for bioequivalence studies [10].

### 2.1. Mechanical Fenestration of Nails

After being surgically removed from cadavers, all hallux nails were gently washed with normal saline to remove any contamination. They were then rehydrated by being placed on a cloth with sterile saline solution (0.9%) for 3 h to flatten the nail. 

**Figure 1:**
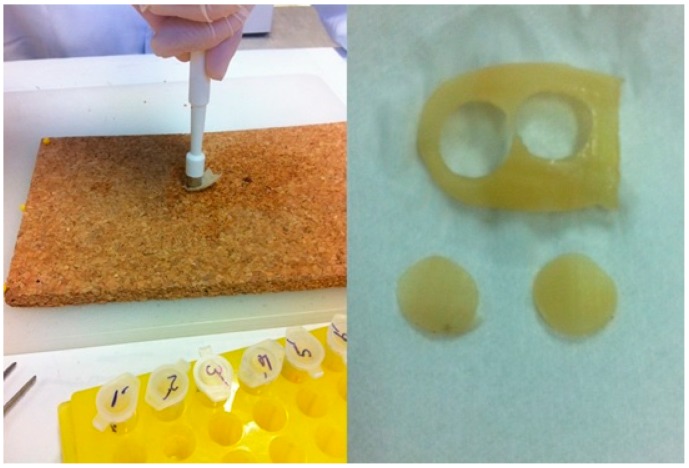
Each hallux nail was punched to obtain two identical discs with a diameter of 0.9 cm. One disc from each nail was then perforated (see below) and one left unperforated.

Following this, each hallux hail was treated with a sterile punching device (0.9 cm in diameter; Medical Train, Madrid, Spain) to obtain two identical 0.9-cm-diameter nail discs derived from each nail. One disc from each nail was added to the “perforated” group and one to the “unperforated” group to minimize variability (Figure 1). The thickness of each hallux nail disc was measured using a digital caliper with a resolution of 0.01 mm and an accuracy of ±0.04 mm (Calibre Digital DCP 500; PCE Iberica S.L., Tobarra, Albacete, Spain).

**Figure 2:**
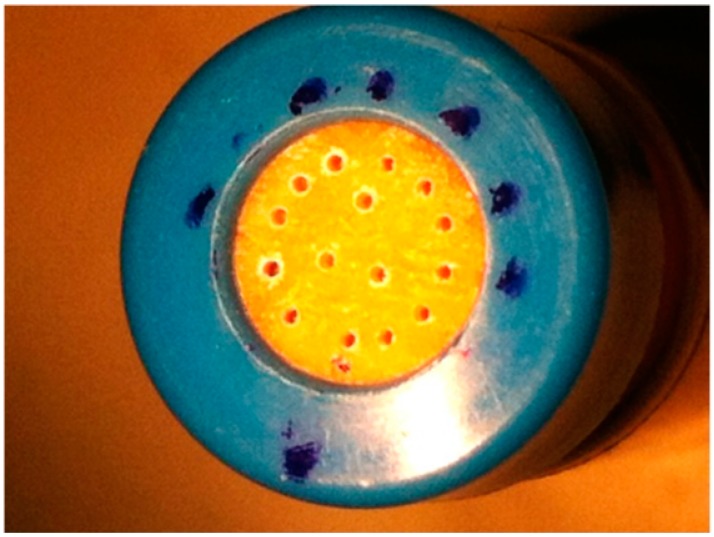
The 12 nail discs of the “perforated nail” toenail group were each drilled with 16 holes using with the *Clearanail^®^* device.

After this, the 12 nail discs in the “perforated” group were each drilled with 16 holes (Figure 2) using the Clearanail^®^ device (Medical Device Treatment Ltd., Brighton, UK; www.clearanail.com), whilst the unperforated group nails were left intact. The Clearanail^®^ device is a nail drill consisting of a motor unit and handle fitted with a single-use 0.4-mm carbide micro cutter. This nail drill is capable of quickly making holes in human nails, but automatically stops once it is through the nail plate to ensure that it does not damage the underlying soft tissue of the nail bed. Holes were drilled only at the start of the study.

### 2.2. Multiple Topical Application Study

Ciclopirox at a concentration of 8% (*w*/*v*) in commercial nail lacquer was applied to all nail discs to examine penetration rate and transungual delivery. Aliquots of 100 µL of the formulation were applied to human toenails every seven days until day 42 when the study ended. Samples were collected from each cell receptor chamber and replaced with a new one before another new drug was applied. This was repeated each time for analysis.

### 2.3. Analytical Method

An in-line diffusion cell (PermeGear, Inc., Hellertown, PA, USA) was used to hold each nail. Nail discs pre-cut to have a diameter of ~0.9 cm were mounted onto the specimen ledge inside the donor chamber of the in-line cell, with the dorsal surface facing the open air. The ventral (inner) surface of the nail was placed face down and rested on the wet cotton pad. To approximate physiological conditions, the cotton pad was wetted with ~0.3 mL of a 6% (*v*/*v*) solution polyethylene glycol (PEG) in a phosphate-buffered saline solution (0.01 M, pH 7.4) before being placed in the receiving chamber to serve as a nail bed and provide moisture for the nail plate. At the defined times, the cotton pad was removed and replaced with a new one in the chamber. Diffusion cells were placed in a water bath (Forma Scientific 2564 CH/P Digital Heated Shaker Water, Marietta, OH, USA) and were set to maintain the temperature at 37 °C and relative humidity at ~55%. Surface washing occurred in the morning at a time that was 10 min prior to next dosing. The nail was washed with cotton tips in the following cycle. A tip was wetted with 0.9% sterile saline solution before this was repeated with distilled water. Washing samples from each cycle of each nail were pooled and collected by breaking off the cotton tip into a glass scintillation vial. Aliquots of 3.0 mL of organic solvent mixture were then added.

### 2.4. Ciclopirox Quantification

The amount of ciclopirox (CPX) in the samples was quantified by high-performance liquid chromatography (HPLC) using previously validated methods [11,12,13]. Two-milliliter HPLC clear glass vials with screw thread closure were used for our study, which had a standard 4.6-mm opening and provided a diffusional area of 0.1662 cm^2^. Glass vials were completely filled with the receptor medium (phosphate-buffered saline solution; pH 7.4). The ventral (inner) surface of the nail disc was placed face down and rested on the liquid surface. Special care was taken to assure that no air bubbles were present between the liquid and the inner nail surface. The nail discs were clamped onto the liquid using the screw thread closure of the vial. 

Once assembled, the glass vials with the nail discs were placed inside a water bath connected to a Haake-DC10 ^®^ circulating bath (Gebruder Haake, Karlsruhe, Germany) to ensure that the system was maintained at a specific working temperature of 37 °C and the surface of the nail plate at a temperature of 32 °C.

A total of 100 µL of CPX lacquer was applied onto the surface of the nail disc. All diffusion experiments were conducted using 12 nail discs. For each glass, 1 mL of the receptor medium was taken every seven days and 1 mL of tempered fresh medium was added to maintain the volume of the receptor chamber. Surface washing was performed onto each disc once the sample was collected. This was achieved using cotton tips wetted with sterile saline solution (washing twice) and a new treatment of 100 µL of CPX lacquer was applied.

**Figure 3:**
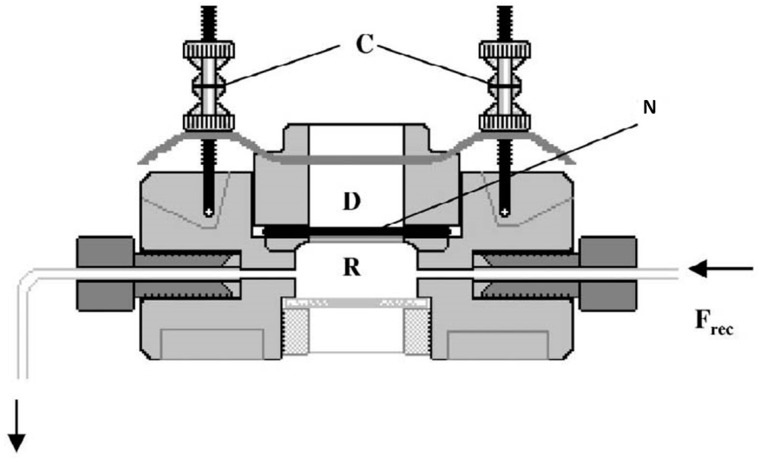
Schematic diagram of a flow-through diffusion cell: D, donor compartment; R, receptor compartment; N, nail; C, clamping system for the donor chamber; Frec, flow of receptor fluid.

A PermeGear ^®^ ILC-07 automated cell diffusion system (PermeGear, Riegelsville, PA, USA) was used to incorporate seven in-line flow-through diffusion cells made of Kel-F, in which the donor and receptor chambers and toenails taken from the foot were clamped by threaded rods with adjustable locking nuts (Figure 3). Each nail disc was placed over a support containing an orifice with a diameter of 0.45 cm, which created a diffusion area of 0.159 cm^2^. The receptor compartment was equipped with a glass viewing window that could be disabled with a locknut. 

The cells were connected to a 16-channel peristaltic pump Ismatec ^®^ IPC-16 (Ismatec, Zurich, Switzerland) and the receptor fluid was pumped continuously through each cell before being collected in the receptor tubes/vials of an Isco^®^ Retriever IV fraction collector (Isco, Lincoln, NE, USA). An Indexing Controller (PermeGear, Hellertown, PA, USA) was used to independently program the duration of each shuttle in the retriever so that 19 samples could be collected simultaneously from each cell.

### 2.5. Statistical Analysis

The means and standard deviations for the primary outcomes were computed. These variables were not normally distributed according to the Shapiro–Wilk test (*p* < 0.05); thus, we reported our results in terms of medians with the respective interquartile ranges.

The thickness of the nail disc followed a normal distribution and, thus, both means and standard deviations were reported with a confidence interval of 95%. A parametric (independent Student’s *t*-test) test was used to detect differences in nail thickness between the two groups of unperforated and perforated toenails.

Repeated measures ANOVA was performed, followed by a Kruskal–Wallis test to determine the significance of any observed differences in the quantities of antifungal agent that passed through the nail discs in the unperforated versus perforated groups.

For all the cases herein, a *p*-value <0.05 with a 95% confidence interval (CI) was considered to be statistically significant. Data analysis was conducted using SPSS software, version 19.0 (SPSS Inc., Chicago, IL, USA).

## 3. Results

The thicknesses of the 12 unperforated and perforated nail discs used in the study did not show any significant statistical differences (*p* = 0.939). The unperforated discs had an average thickness of 0.99 ± 0.05 (95% CIs = 0.96–1.02) while the perforated discs had an average thickness of 0.99 ± 0.05 (95% CIs = 0.96–1.04) mm. The normal distribution of the studied variables is shown in Table 1.

The penetration of 8% ciclopirox was determined every seven days until day 42 in all perforated and unperforated nail discs. There was a linear increase of ciclopirox recovered in all samples (see Table 2), but penetration was significantly higher in perforated nail discs at each time point. The total transungual ciclopirox recoveries were 2067.85 ± 1216.34 µg (95% CIs = 851.51–2756.05) and 540.32 ± 590.21 µg (95% CIs = 206.38–874.26) µL (*p* < 0.008) for the perforated and unperforated nail disc groups, respectively.

## 4. Discussion

The focus of this study was to evaluate the effectiveness of nail fenestration in promoting penetration of antifungal agents through the nail, while not having an impact on the efficacy of the drug. Ciclopirox was chosen because the posology of this agent requires a weekly application of the agent. 

In the present study, we determined that the amount of ciclopirox in the transungual region increased linearly with time in both groups of nail discs, but was significantly (~3–4-fold) higher in perforated toenails after 42 days of treatment.

Normally, when a drug is applied topically to human nails, compounds may accumulate in the dorsal nail surface layer and this impedes its penetration to the deep ventral layer and the bed [3,5,14]. By introducing holes into the overlying plate, an antifungal solution can readily reach the point of infection directly with little or no reduction in its effectiveness. This may account for the rapidity in the action of antifungals in the previous reports [8]. 

To our knowledge, this study represents a novel quantitative in vitro model designed to investigate the efficacy of transungual drug penetration using a novel mechanical modality to perforate the nail. 

The concept of rendering the nail more permeable to topical agents is not a new one. Many physical and chemical treatments [15,16] underwent testing to determine their ability to increase nail permeability and drug penetration, including physical methods (iontophoresis, etching, lasers, ultraviolet (UV) light, and phonophoresis), chemical methods (mercaptans thiols, keratolytic agents, and organic solvents), mechanical methods (nail abrasion and nail avulsion), enhanced drug delivery (electro-chemotherapy), and mesoscissioning technology [15].

However, most of these treatments are laborious, require extensive training, or are expensive. Furthermore, many need specialist equipment, limiting their use in treatment practice. Nail drilling would be much easier to deploy.

The technology employed in the Clearanail^®^ and similar devices permits safe nail-plate drilling. The system detects the difference in the nail plate and nail bed by monitoring the power required to rotate the drill bit. As nail keratin is harder than the underlying epidermis, more power is required by the drill when penetrating the harder layers. As soon as softer underlying structures are reached, the power demand of the cutter diminishes, triggering the cessation of drilling. Holes within the nail plate remain present for the duration of treatment before eventually growing out as nail growth advances. Thus, nail fenestration using intelligent nail-drilling devices appears to be a promising avenue for future treatment regimens designed to combat onychomycosis.

Certain diseases, such as onychomycosis, can produce hyperkeratosis or alterations of the shape of the nail plate that may influence the penetration of topical medicaments. Accordingly, one limitation of this study is that the toenails used in the research were healthy nails; further research is needed in toenails affected by onychomycosis.

## 5. Conclusions

The fenestration of the nail plate prior to the application of topical antifungal agents was previously shown to be an effective treatment [8]. This present study demonstrates that nail fenestration increases transungual delivery of the antifungal agent ciclopirox by 3–4-fold over unperforated controls. It is likely that fenestration would benefit all topical antifungal agents and might increase the range of agents and formulations that can be used to treat onychomycosis in the future. The in vitro model described herein would be an excellent system to directly measure transungual delivery of antifungal agents prior to clinical trials. The current in vitro data presented in this study, together with previous treatment data [8], appear to justify a clinical examination of fenestration of the toenail as a possible treatment method for onychomycosis.

## Figures and Tables

**Table 1:** Normality distribution of the data. CI—confidence interval.

Variables	Unperforated Toenail (*n* = 12)	Perforated Toenail (*n* = 12) Mean ± SD (95% CIs)
Nail thickness	0.286	0.228
7 days	<0.001	0.349
14 days	<0.001	0.291
21 days	<0.001	0.146
28 days	0.001	0.087
35 days	0.008	0.085
42 days	0.010	0.060
Total amount recovered	0.002	0.081

Shapiro–Wilk test; *p* < 0.05 (with a 95% confidence interval) was considered to be statistically significant.

**Table 2:** Total transungual ciclopirox (µg) recovered in unperforated and perforated nail discs at various time points after the beginning of the experiment.

Day	Unperforated Toenails (*n* = 12) Mean ± SD (95% CIs)		Perforated Toenails (*n* = 12) Mean ± SD (95% CIs)		*p*-Value
	Mean ± SD (95% CIs)	Median (interquartile range)	Mean ± SD (95% CIs)	Median (interquartile range)	
7	24.70 ± 43.35 (0.17–49.22)	0.67 (52.81)	105.05 ± 87.24 (17.81–154.41)	108.86 (172.59)	0.004
14	43.38 ± 73.93 (1.55–85.21)	2.15 (98.46)	208.37 ± 152.43 (55.94–294.62)	167.62 (264.69)	0.002
21	57.04 ± 92.94 (4.45–109.63)	8.34 (136.59)	289.26 ± 204.01 (85.25–404.69)	236.71 (406.17)	0.001
28	76.35 ± 107.96 (15.26–137.43)	20.46 (147.05)	388.34 ± 225.31 (163.02–515.82)	341.96 (446.75)	0.001
35	139.18 ± 140.57 (59.65–218.72)	75.77 (209.19)	483.78 ± 249.76 (234.02–623.10)	453.08 (503.12)	0.001
42	199.65 ± 148.65 (115.54–283.75)	126.73 (227.99)	593.02 ± 335.21 (257.80–782.68)	460.85 (635.67)	0.001
Total amount	540.32 ± 590.21 (206.38–874.26)	223.34 (870.04)	2067.85 ± 1216.34 (851.51–2756.05)	1848.18 (2427.01)	0.001

A Kruskal–Walis test was performed (*p* < 0.05) and these data were considered to be statistically significant at the 95% confidence interval.
